# Missed opportunities for NCD multimorbidity prevention in adolescents and youth living with HIV in urban South Africa

**DOI:** 10.1186/s12889-020-08921-0

**Published:** 2020-06-01

**Authors:** Monika Kamkuemah, Blessings Gausi, Tolu Oni

**Affiliations:** 1 Research Initiative for Cities Health and Equity, Division of Public Health Medicine, School of Public Health and Family Medicine, University of Cape Town, Cape Town, 7925 South Africa; 2 Medical Research Council Epidemiology Unit, University of Cambridge, Cambridge, CB2 0QQ UK

**Keywords:** HIV, Non-communicable Disease, Adolescents, Youth, Multimorbidity

## Abstract

**Background:**

Epidemiological transition in high HIV-burden settings is resulting in a rise in HIV/NCD multimorbidity. The majority of NCD risk behaviours start during adolescence, making this an important target group for NCD prevention and multimorbidity prevention in adolescents with a chronic condition such as HIV. However, there is data paucity on NCD risk and prevention in adolescents with HIV in high HIV-burden settings. The aim of this study was to investigate the extent to which NCD comorbidity (prevention, diagnosis, and management) is incorporated within existing adolescent HIV primary healthcare services in Cape Town, South Africa.

**Methods:**

We reviewed medical records of 491 adolescents and youth living with HIV (AYLHIV) aged 10–24 years across nine primary care facilities in Cape Town from November 2018–March 2019. Folders were systematically sampled from a master list of all AYLHIV per facility and information on HIV management and care, NCDs, NCD risk and NCD-related health promotion extracted.

**Results:**

The median age was 20 years (IQR: 14–23); median age at ART initiation 18 years (IQR: 6–21) and median duration on ART 3 years (IQR: 1.1–8.9). Fifty five percent of participants had a documented comorbidity, of which 11% had an NCD diagnosis with chronic respiratory diseases (60%) and mental disorders (37%) most common. Of those with documented anthropometrics (62%), 48% were overweight or obese. Fifty nine percent of participants had a documented blood pressure, of which 27% were abnormal. Twenty-six percent had a documented health promoting intervention, 42% of which were NCD-related; ranging from alcohol or substance abuse (13%); smoking (9%); healthy weight or diet (9%) and mental health counselling (10%).

**Conclusions:**

Our study demonstrates limited NCD screening and health promotion in AYLHIV accessing healthcare services. Where documented, our data demonstrates existing NCD comorbidity and NCD risk factors highlighting a missed opportunity for multimorbidity prevention through NCD screening and health promotion. Addressing this missed opportunity requires an integrated health system and intersectoral action on upstream NCD determinants to turn the tide on the rising NCD and multimorbidity epidemic.

## Background

### Emerging NCD epidemic in low and middle-income countries

Non-communicable diseases (NCDs) are responsible for more than half of the global burden of disease [1] with 71% of global deaths in 2016 due to NCDs [2]. The burden of NCDs is increasing most rapidly in low- and middle-income countries (LMIC) with NCDs projected to account for nearly half of the burden of disease in low-income countries by 2030 and a four-fold higher probability of NCD-related premature deaths compared to high-income countries [2]. This trend is driven by rapid unplanned urbanization, globalization of unhealthy lifestyles such as tobacco use, physical inactivity, unhealthy diet and harmful use of alcohol [3] and population ageing [4, 5], resulting in a protracted epidemiological transition [6] with an emerging epidemic of obesity, diabetes and other NCDs alongside persisting infectious disease epidemics [7].

Of note, NCDs are affecting younger populations, and are associated with worse health and economic outcomes in low- and middle-income countries compared to high-income countries [8]. The estimated overall cost of diabetes in sub-Saharan Africa was US$19·45 billion or 1·2% of cumulative gross domestic product in 2015 [9] with health systems unable to provide adequate management for diabetes and its associated risk factors and sequelae [9]. This growing cost of NCDs is of particular concern as LMICs are grappling with higher levels of NCD at earlier stages of economic development, with fewer resources, and with less time to respond effectively [10]. This highlights the importance of a focus on prevention of NCDs through early identification of modifiable risk factors at the primary care level across the life-course as the most feasible and cost- effective approach to maintaining population health [11].

### NCD prevention in adolescents

Adolescence, a period of transition from childhood to adulthood, is defined as the period between the ages of 10–19 years [12], with some definitions extending this life stage to 24 years [13]. It is a period that comes with unique challenges as adolescents strive to develop autonomy and self-identity through exploration and risk-taking [14–16], with a significant part of adolescence lying in its power to translate childhood experiences into competencies and statuses that facilitate the transition to adulthood [16].

Adolescents and youth aged 10–24 years make up over 25% of the global population, and their numbers are set to rise to two billion by 2032 [17]. Africa has the youngest population in the world, with more than one-third of the total population of sub-Saharan African aged 10 to 24 years [18]. Although adolescents are generally healthy, up to 70% of premature adult deaths reflect behaviours started or reinforced during adolescence [19]. Worldwide, over 10% of young people smoke [20], 81% of adolescents have insufficient physical activity; and 11.7% of adolescents partake in heavy episodic drinking [3, 21]. This is further shaped by the social and commercial determinants of health which influence health-related behaviours. Reinforcing healthier behaviours and protective factors during this critical period of adolescence, can significantly change the health trajectory into adulthood [22].

### The rise of multimorbidity in the context of HIV

With the introduction of antiretroviral therapy (ART), HIV can be considered a chronic disease [27]. In countries with a high burden of HIV undergoing rapid urbanization and epidemiological transition, HIV/NCD multimorbidity is becoming increasingly prevalent [25] as people are living longer with HIV [23, 24]. HIV-infected individuals are at increased risk of NCDs compared to HIV-uninfected persons, due partly to HIV-infection [26, 27] and the complications of long-term anti-retroviral therapy [28–30]. A study in the United States reported that HIV-infected adults had a 1.5 fold increased risk of having a heart attack [31] and were nearly 50% more likely to have received a diagnosis of chronic obstructive pulmonary disease (COPD) compared to demographically and behaviourally similar uninfected controls [32]. Other NCDs more commonly reported in HIV-infected compared to un-infected persons include cardiovascular events [33], hypertension [34, 35], osteoporosis [36], renal impairment [37], diabetes mellitus [38], and depression and neurocognitive disorders [39].

In high HIV-burden settings, multimorbidity is occurring at younger ages with younger persons at a higher level of risk for heart-related conditions attributed to HIV infection [27]. HIV-infected adolescents, particularly those with increased HIV viral load, have been reported to have increased levels of cholesterol and triglycerides [40] and increased biomarkers of vascular dysfunction than HIV-exposed, uninfected adolescents [41]. This is concerning for LMICs where there is a younger age distribution of HIV-infected populations [27]. In a review by Lowenthal et al., the authors found that in sub-Saharan Africa, one of the striking features of HIV infection in adolescence is the high prevalence of chronic complications like chronic lung disease, cardiac disease, growth failure, neurocognitive disease, skin, renal and bone diseases [42].

This trend, and the fact that many unhealthy behavioural patterns develop in adolescence, make integrated prevention efforts necessary, and highlight the importance of a focus on HIV-infected adolescents and youth for multimorbidity prevention.

### Adolescents, HIV and the epidemiological transition in South Africa

South Africa has the largest HIV epidemic worldwide and the largest ART programme in the world. In 2016, an estimated seven million people were living with HIV in South Africa, with four million of those receiving ART [43]. This epidemic persists in the context of epidemiological transition characterised by a quadruple burden of communicable, non-communicable, perinatal and maternal, and injury-related disorders [44]. Of the 1.6 million adolescents (aged 10–19 years) living with HIV globally in 2018, 310,000 (19%) are in South Africa [45]. These adolescents are growing up in an era of epidemiological transition, characterised by increasing burden of NCDs driven by the unhealthy environments within which they live, necessitating interventions to enhance protective environmental factors and minimize risk behaviours [46].

### The opportunity for NCD prevention in adolescents with a chronic disease (HIV)

Whilst young people generally do not access health care services regularly, living with a chronic condition like HIV which requires regular interaction with the health care system [47] represents an opportunity for early identification of NCD risk and intervention to reduce the risk of NCD.

In South Arica, HIV patients in the public sector are primarily seen and treated in HIV clinics. They are routinely monitored via physical examination, point of care and laboratory assessments as part of routine clinical management for HIV. This includes general health screening, patient history and biomedical assessments which can also be used for other chronic disease screening. There has been a gradual move away from vertical services within HIV care, towards more integrated care that includes NCD screening and management in low- and middle-income countries which face a dual burden of HIV and NCDs. An integrated approach which harnesses the successful programmatic approach of the HIV treatment program is recommended to scale up services for NCDs [48]. In South Africa, the National Health Department introduced the Integrated Chronic Disease Management (ICDM) model into primary health care facilities in 2011 in order to bridge fragmented chronic disease care within the public health care system and improve chronic disease health outcomes [49, 50]. However, to date, these efforts have focused on adults. This is despite a recognition of an increasing burden of NCDs among people living with HIV occurring at increasingly earlier ages [25, 27, 51], and the importance of the adolescent and early youth period for development of NCD risk factors that increase the risk of NCDs [13, 14].

Although systematic NCD screening is not formally integrated within adolescent primary care HIV services, general health screening and laboratory tests are done to identify biomedical risk factors. However, it is unclear the extent to which these preventive activities are conducted. We therefore set out to investigate the extent to which NCD comorbidity (prevention, screening and management) is incorporated within existing adolescent HIV primary healthcare services in Cape Town, South Africa.

## Methods

### Setting

#### Cape Town- demographics and epidemiology

The study was conducted in Cape Town, the second most populous city in South Africa with an estimated population of 3.8 million people [52]. In 2016, adolescents aged 10–19 years comprised 16.1% of the population in the Western Cape province, within which Cape Town is located [53].

Data for this study was collected from public primary health care facilities across Cape Town: Khayelitsha, Gugulethu, Langa, Brooklyn, Michell’s Plain, Kraaifontein and Delft. These peri-urban neighbourhoods, located on the outskirts of Cape Town are comprised of a mix of formal and informal dwellings (with 20.5% of the population residing in informal dwellings [54]), are characterised by high unemployment levels (30%), with approximately 63% of households in the Khayelitsha/Mitchells Plain district falling within the low income bracket (earning < 280 USD per month) [55].

Non-communicable diseases and HIV are ranked amongst the top causes of premature deaths in the City of Cape Town [54], with the top five causes of death in the Western Cape in 2016 ranked as diabetes mellitus, HIV, ischaemic heart diseases, cerebrovascular diseases and tuberculosis [56]. Antenatal HIV prevalence in Cape Town city was estimated at 21.6% in 2015 [57], and as high as 34.3% in Khayelitsha in 2012 [58].

#### Health system and HIV/NCD healthcare delivery

The City of Cape Town Metro Health district has eight legislated sub-districts. Primary health care services are delivered through four sub-structures (comprised of two sub-districts in each sub-structure): Khayelitsha/Eastern, Mitchell’s Plain/ Klipfontein, Western/Southern and Northern Tygerberg sub-districts [54]. ART care is delivered through government clinics and is free to all patients, with patients with well-controlled disease managed in ART “clubs”, which are either facility- or community-based, and provide health screening, more streamlined access to repeat prescriptions, health promotion, education and socialisation for patients. There were 5723 children under 15 years receiving ART in the public health system in Cape Town in November 2016 (personal communication with Director of HIV Treatment Programme: Provincial Government of the Western Cape).

NCD screening, prevention and care is provided at the primary care level as an entry point, with routine clinical management of NCDs in primary care outpatient clinics. Within these clinics, management of NCD patients with well-controlled disease is primarily through chronic disease “clubs” in a similar manner to patients on ART.

### Study design and sampling

We conducted a descriptive retrospective study from November 2018 to March 2019, reviewing clinic folders of adolescents and youth enrolled in HIV care across Cape Town.

#### HIV clinic selection

The total number of children under 15 years receiving HIV care in all clinics across all four legislated sub-structures, was extracted from an internal report provided by the Western Cape Department of Health. As there were no routinely collected data for adolescents, we utilised data for children under 15 years as a proxy for adolescence and to identify healthcare facilities with the largest number of adolescent and youth populations in care. Clinics with the highest number of children under 15 registered as of November 2016 were selected and a request for access to these submitted to the Western Cape Department of Health. Approval was received for nine clinics with the busiest 1–3 clinics selected from each sub-structure.

#### Folder selection within each clinic

**Fig. 1:**
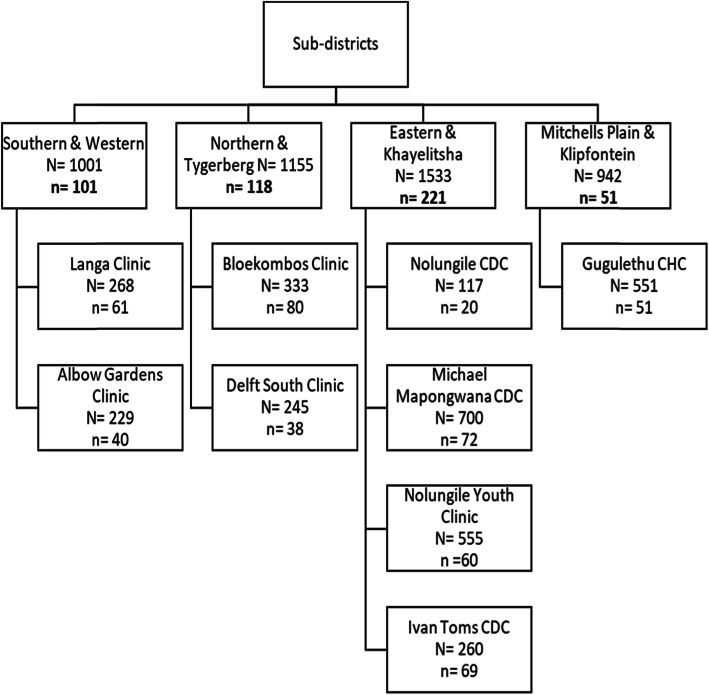
Study population and sampling methodology across sub-districts

At each clinic selected, a de-identified population master list of all adolescents and youth aged 10–24 years receiving ART was generated by visiting each facility and compiling a sampling frame of 10–24 year old patients with the assistance of data clerks at each facility. We aimed to sample 10% of adolescents on ART from each sub-structure (*n* = 463 overall) with the required sample size at each clinic in each substructure calculated proportional to the denominator of the total adolescent patient population (Fig. 1). Systematic random sampling was used to select folders from the sampling frame master list, with the first folder selected from the population list at random, and every *nth* folder sampled thereafter, where the sampling interval *n* was determined by the number of adolescents in the clinic population divided by the number of adolescents needed for the sample. In the event the *nth* folder was not found, the next folder in the list was selected and the systematic sampling continued until 10% of folders of adolescents in each sub-structure were reviewed. Data collection was conducted between November 2018–March 2019.

### Data extracted from folders

Health care visits in the preceding 12 months were reviewed in each folder. In patients that had another chronic disease diagnosis recorded, all episodes recorded in the folder, irrespective of date, were extracted.

Data relating to HIV including treatment and opportunistic infections, NCD diagnosis and treatment (diabetes, hypertension, asthma, mood disorders, epilepsy), NCD risk (overweight/obesity, raised blood pressure, substance abuse, and smoking), and general medical history including family history, were extracted from participant folders (See Table 1). Any recorded health promotion interventions related to HIV, sexual health or NCD risk were also extracted. A more detailed description of data collected and definitions used is included in an additional file [see Additional file 1].

**Table 1:** Demographic and HIV-related characteristics of 491 participants aged 10–24 years receiving ART across Cape Town, November 2018–March 2019

	Characteristic	*N* = 491^a^	Median (IQR) or n (%)^b^
**Demographics**	Age at folder review (years)		20 (14–23)
	Male		127 (26%)
**HIV-related**			
HIV management	Age at ART initiation (years)	473 (96%)	18 (6–21)
	Duration on ART (years)	471 (96%)	3.1 (1.1–8.9)
	CD4 count (cells/mm^3^)	395 (80%)	489 (355 --690)
	Viral suppression (< 20 copies/ml)	369 (75%)	256 (69%)
Current ART regimen^c^	**First line:** ABC 3TC EFV		84 (17%)
	TDF FTC EFV		298 (61%)
	**Second line:** ABC 3TC LPV/r		39 (8%)
	AZT 3TC LPV/r		29 (6%)
	Other regimens		41 (8%)
HIV opportunistic infections		28 (6%)	
	Herpes zoster (shingles)		11 (39%)
	Pruritic papular eruption		8 (29%)
	Oral thrush		2 (7%)
	Oral candida		2 (7%)
	Oral hairy leucoplakia		1 (3.6%)
	Oesophageal candidiasis		1 (3.6%)
	*Pneumocystis jirovecii* pneumonia		1 (3.6%)
	HIV Encephalopathy		1 (3.6%)
	Cytomegalovirus disease		1 (3.6%)
ART-related conditions^d^		8 (2%)	
	AZT-neutropenia		1 (13%)
	D4T-lipodystrophy, lipoatrophy		7 (87%)

^a^Denominator = 491, unless otherwise indicated in this column; ^b^Percentages may not total 100 due to rounding; ^c^Current ART regimens: *ABC* Abacavir, *3TC* lamivudine, *EFV* efavirenz, *TDF* tenofovir, *FTC* emtricitabine, *LPV/r* lopinavir/ritonavir; ^d^ART-related conditions: *AZT* zidovudine, *D4T* stavudine

### Analysis

Descriptive statistics (median (IQR) and frequencies and percentages) were used to describe continuous and categorical variables, respectively. All analyses were conducted using STATA v14.0 (*StataCorp. 2015. Stata Statistical Software: Release 14. College Station, TX: StataCorp LP*).

## Results

A total of 491 folders were reviewed from a master list of 4631 patients. The median participant age was 20 years (Interquartile range (IQR): 14–23 years); 74% were female; median age at ART initiation was 18 years (IQR: 6–21 years) and the median duration on ART was 3 years (IQR: 1.1–8.9 years) (Table 1).

### HIV management

The majority of patients were virally suppressed (69%) (median CD4 count 489 cells/mm^3^ (IQR (355—690 cells/mm^3^)) and on first line ART regimens (78%), (fixed-dose tenofovir-based (61%); abacavir- lamivudine based regimens (17%)) as shown in Table 1.

### HIV opportunistic infections

Seven percent (36/491) had a documented HIV opportunistic infection or an ART-related condition. Of those, the most prevalent conditions were herpes zoster/ shingles (39%), followed by pruritic papular eruption (PPE) (29%), and oral thrush (7%) or oral candida (7%). The reported cases of opportunistic infections were documented to have received appropriate treatment (antibiotics, antivirals or antifungals). In addition, 14% of non-ARV medications prescribed in the previous 12 months consisted of cotrimoxazole prophylaxis to prevent opportunistic infections.

### NCD comorbidity

Fifty-five percent (*n* = 268) of folders reviewed had documented information on comorbidities, of which 11% were NCD comorbidities. Of these, the most prevalent NCDs documented were chronic respiratory diseases (asthma, bronchitis, COPD (60%)) and mental health disorders (depression, anxiety or other mental health conditions (37%)) (Table 1). Despite this, only one participant was documented as receiving treatment for asthma. Other documented NCD treatment received in the previous 12 months were for high blood pressure, psychosis and high cholesterol/ triglycerides.

### NCD risk

In terms of NCD risk factors documented, 4% were current smokers or had a history of smoking, and 3% used alcohol, drugs or other substances. Only 62% of folders reviewed had documented anthropometric data (height and weight). Of these, 48% were overweight or obese (26 and 22% respectively) and 10% were underweight. Fifty-nine percent of folders reviewed had a documented blood pressure. Of these, the majority (73%) were normal (< 130/85 mmHg), 14% had elevated blood pressure, and 12% showed signs of mild hypertension (SBP 140-159 mmHg or DBP 90- 99 mmHg). There were three cases (1%) with moderate hypertension documented (SBP 160–179 mmHg or DBP 100–109 mmHg).

### General medical information

Family history was documented in 6% of the folders reviewed. Of these, the most common condition documented was tuberculosis (69%), followed by diabetes (14%), high blood pressure (7%) and alcoholism (7%). Other non-infectious conditions were documented in 11% of the folders reviewed. These conditions ranged from current/ previous pregnancy (60%), experiences of trauma due to injury or violence (11%) and epilepsy, learning difficulties (10%) and failure to thrive (10%). There were isolated cases reported of conditions such as peripheral neuropathy, hearing loss, impetigo, severe dermatitis and lymphadenopathy.

### Non-HIV infectious diseases

Thirty-eight percent had non-HIV infectious diseases on record. Tuberculosis was the most documented infectious disease. Of the 38% with an infectious comorbidity reported, 62% had been diagnosed with tuberculosis in the past, 22% had been diagnosed with a sexually transmitted infection and 6% had a history of scabies. Five percent had been diagnosed with pneumonia as an infant or continued to experience severe recurrent bacterial/ viral pneumonia (not *Pneumocystis jiroveci pneumonia-*PJP). Herpes simplex virus and meningitis were reported in 3% of the folders.

Other non-ARV medications prescribed in the last 12 months (27%) were TB prophylaxis (22%), antibiotics (33%), STI treatment (17%), steroids (12%), antiviral medication (2%) and contraceptives (4%).

### Health promotion interventions

Twenty-six percent of participants had a documented health promoting intervention, ranging from HIV- and NCD- to sexual and reproductive health-related interventions. Seven percent received disclosure counselling to facilitate full disclosure of their HIV status and 9% underwent adherence counselling (Table 2). For NCD-related health promotion, 13% received alcohol or substance abuse counselling; 10% received mental health counselling, 9% were advised on healthy weight or diet and 9% were counselled about smoking tobacco. One singular case was documented of a diabetes screening intervention. Eleven percent underwent family planning or basic antenatal care counselling each, while 5% were referred for a pap smear or breast examination, Medical male circumcision and safe sex counselling were documented in 2% of the folders. Other health promotion interventions documented were hygiene counselling (3%) and physiotherapy/occupational therapy (2%).

**Table 2:** NCD comorbidity, general medical information and health promotion interventions documented in folders of 491 participants aged 10–24 years receiving ART across Cape Town, November 2018–March 2019

	Characteristic	*N* = 491^a^	n (%)^b^
**Comorbidity Information**		268 (55%)	
NCD diagnosis		30 (11%)	
	Depression, anxiety or other mental health condition		11 (37%)
	Bronchitis, lung disease, asthma or other chronic respiratory disease^c^		18 (60%)
	Cancer		1 (3%)
NCD treatment		4 (1%)	
	Asthma treatment		1 (25%)
	High Blood Pressure treatment		1 (25%)
	Antipsychotic medication		1 (25%)
	High cholesterol/ triglycerides		1 (25%)
NCD risk factors		305 (62%)	
	Smoking: current smoker or history of smoking		11 (4%)
	Alcohol, drugs or other substance abuse		9 (3%)
Body Mass Index (BMI) kg/m^2^		305 (62%)	
	Underweight: BMI < 18.5		30 (10%)
	Normal weight: BMI 18.5–25		129 (42%)
	Overweight: BMI 25–30		80 (26%)
	Obese: BMI ≥30		66 (22%)
Blood Pressure mmHg^d^		289 (59%)	
	Normal: SBP < 130 and DBP < 85		210 (73%)
	High normal: SBP 130–139/DBP 85–89		41 (14%)
	Mild hypertension: SBP 140–159/DBP 90–99		35 (12%)
	Moderate hypertension: SBP 160–179/ DBP 100–109		3 (1%)
**General medical information:** Contraception			18 (4%)
Family History		29 (6%)	
	Tuberculosis		20 (69%)
	Diabetes		4 (14%)
	High blood pressure		2 (7%)
	Alcoholism		2 (7%)
	Cancer		1 (3%)
Other conditions		52 (11%)	
	Pregnancy		31 (60%)
	Trauma- injury and violence		6 (11%)
	Epilepsy		5 (10%)
	Learning Difficulties		5 (10%)
	Failure to Thrive		5 (10%)
**Non-HIV infectious diseases**		185 (38%)	
Non-HIV infectious disease diagnosis	Tuberculosis		114 (62%)
	Sexually Transmitted Infections		40 (22%)
	Scabies		12 (6%)
	Pneumonia		9 (5%)
	Herpes Simplex Virus		5 (3%)
	Meningitis		5 (3%)
**Non-ARV medications prescribed in Iast 12 months**		135 (27%)	
	TB prophylaxis		30 (22%)
	Cotrimoxazole		19 (14%)
	STI treatment		23 (17%)
	Antibiotics		44 (33%)
	Steroids		16 (12%)
	Antivirals		3 (2%)
**Health Promotion**		93 (19%)^e^	
HIV-related	Disclosure counselling		9 (7%)
	Adherence counselling		11 (9%)
NCD-related	Alcohol counselling		14 (11%)
	Mental health counselling		13 (10%)
	Healthy diet or weight counselling		12 (9%)
	Smoking counselling		11 (9%)
	Substance abuse counselling		2 (2%)
	Diabetes screening		1 (1%)
Sexual and Reproductive Health	Family planning		14 (11%)
	Basic antenatal counselling		14 (11%)
	Postnatal care & Infant feeding counselling		10 (8%)
	Pap smear & Breast examination		6 (5%)
	Safe sex counselling		2 (2%)
	Medical male circumcision		2 (2%)
Other Health Promotion	Hygiene counselling		4 (3%)
	Physiotherapy/Occupational Therapy		2 (2%)

^a^Denominator = 491, unless otherwise indicated in this column

^b^Percentages may not total 100 due to rounding

^c^Includes asthma, Chronic Obstructive Pulmonary Disease, and other respiratory conditions

^d^SBP = Systolic Blood Pressure and DBP = Diastolic Blood Pressure

^e^Total interventions > 93 as some individuals had more than one form of health promotion documented

## Discussion

This study describes documentation of NCD and NCD risk screening and health promotion in HIV-infected adolescents and youth receiving ART in an urban setting in South Africa. We found that only 55% of the folders reviewed had any information on other comorbidities and 62% had risk factor information. Of these, 11% were NCD comorbidities ranging from mental health conditions to chronic respiratory diseases. A key finding of this study is the paucity of data on NCD and NCD risk captured as part of clinical care of adolescents with HIV. Poor documentation and screening of NCD risk-factors for the majority of participants in our study demonstrates a missed opportunity for detecting comorbidity and NCD risk in primary health care and for early intervention in AYLHIV who represent an important population and are less inclined to seek regular or preventive care. Early identification and intervention to modify behaviour would prevent a costly future epidemic of NCDs and avert morbidity and mortality due to NCDs [10].

Data paucity notwithstanding, our results highlight evidence of co-existing NCD multimorbidity and NCD risk factors (overweight and obesity, elevated BP and smoking, alcohol and substance use) in AYLHIV. A similar study in the US conducted a retrospective chart review in HIV-positive children and adolescents aged 2–25 years and found an 18% prevalence of high blood pressure [64]. In that study, there were significant associations with other medical comorbidities and risk factors such as tobacco exposure and male gender. The authors highlighted that the life-long cardiovascular risks associated with HIV infection and its management call for closer monitoring and possibly treatment of elevated BP in this population [64]. Another study conducted in Cape Town adults in similar peri-urban informal settings as our study demonstrated that 19% of HIV-infected patients on ART were on treatment for another chronic disease (diabetes, tuberculosis or hypertension), with 77 and 17% of them receiving anti-hypertensive and diabetic treatment respectively [25].

Previous studies in healthy young people have shown prevalence rates of overweight and obesity of 23 to 7% respectively [65] and hypertension/elevated blood pressure rates of 6.7% in respondents in the 15–24-year age group [59]. Whilst the prevalence of these NCD risks cannot be estimated from our study due to the limited documentation, these previous surveys in South Africa demonstrate risk factors for NCDs in adolescents in the general population. Given the data from adults with HIV in South Africa, there is an indication that the prevalence of NCD risk in adolescents with HIV is potentially higher than their healthy counterparts [25, 66], strengthening the argument for targeted NCD prevention efforts in this population group to prevent multimorbidity. Given that some NCDs (such as mental disorders) and many NCD risk behaviours such as substance abuse also influence HIV control, our finding that 69% of participants were virally suppressed further emphasises the need for strengthened integrated health systems.

In this study, we noted that only 19% had a documented health promoting intervention, ranging from alcohol or substance abuse (13%) to healthy weight or diet (13%) and mental health counselling (10%). Family history of an NCD has been shown to be a significant risk factor for NCD in South Africa [59] and so should form an important component of NCD risk assessment. In this study, only 6% had a documented family history recorded. Other upstream determinants of NCD risk such as the social environment were not noted.

As has been demonstrated in adult patients, chronic disease care requires a comprehensive, holistic approach that integrates treatment and prevention of multiple conditions [60, 61]. Such an approach, integrating NCD primary prevention with HIV care, will be an important component of strategies to reduce multimorbidity and the future burden of NCDs in high HIV-burden settings. In South Africa, strategies like the ICDM and Chronic Disease Clubs, responding to the observed epidemiological transition and rise of HIV/NCD multimorbidity, are aimed at integrating chronic (infectious and non-communicable) disease programs using established and existent frameworks to expand access to primary care that includes services for both HIV and NCDs [48].

Pilot projects are underway in selected primary health care facilities to investigate the most effective models of integrated care [62]. Early findings demonstrate provider and patient satisfaction with several dimensions of the model [63]. But leveraging elements of HIV programmes for NCDs, like hypertension management was noted to be inadequate, in part due to malfunctioning equipment and drug stock-outs [62]. To date, these models have focused on the general adult population, with no integrated clinics for adolescents planned largely due to a paucity of data on NCD co-morbidities and NCD risk in adolescents with HIV. These efforts have largely ignored adolescents and youth with a focus solely on HIV-specific outcomes and neglecting an opportunity to intervene holistically in a target at-risk population regularly accessing care.

Our results demonstrate a missed opportunity to improve health, and prevent multimorbidity, in this important population group with unique health needs.

A key limitation of our study was the retrospective nature of data collection. We were unable to estimate the prevalence of NCD or NCD risk in this population due to the possibility of screening bias and underreporting. Furthermore, the health promotion activities assessed are those which were documented in patient folders which may under-estimate actual health promotion interventions delivered. Given the limitations of the study design, we instead set out to describe the extent to which NCD comorbidity screening, prevention and management is incorporated within existing adolescent HIV primary healthcare services.

Another limitation is that we were unable to explore determinants of NCD comorbidity due to the low number of NCD diagnoses recorded. Such information could be used to inform targeted and cost-effective approaches to NCD screening.

However, the NCD data paucity noted represents an important finding for the health system as it demonstrates limited consideration of NCD prevention in HIV care and highlights a missed opportunity for NCD prevention in a patient group regularly accessing health care. This study was therefore an important first step to inform future research on the epidemiology of NCD and NCD risk factors in AYLHIV.

## Conclusion

Our data demonstrates the existence of NCD risk factors in adolescents and youth, though poorly documented at the primary care level. This highlights a missed opportunity in multimorbidity prevention through the provision of NCD screening and prevention services to AYLHIV. Further research is needed to better ascertain NCD prevalence and NCD risk epidemiology in AYLHIV. Whilst our study focused on HIV, these findings are relevant for adolescents with any chronic condition who are interacting with health services regularly.

Addressing this missed opportunity would require an integrated health system. Further research is needed to inform the most effective models of care for HIV management and integrated NCD prevention in order to effectively respond to communicable and NCD prevention and control. In addition, intersectoral collaboration with non-health sectors incorporating upstream environmental, socio-economic and cultural determinants of NCD risk into prevention efforts are vital to multimorbidity prevention efforts, particularly in the context of rapid urbanization. An early identification and prevention approach to NCD control in HIV-infected adolescents and young adults is vital to turn the tide on the NCD and multimorbidity epidemic and avert the economic implications of NCDs to individuals, families and societies, whilst simultaneously improving HIV outcomes and reducing the risk of NCD in this key population group.

## Supplementary information

**Additional file 1.** contains detailed information on all data collected as part of the folder review process and definitions of variables used in the study.

## Data Availability

The data that support the findings of this study are not publicly available due to the sensitive nature of information that could compromise minor research participants’ privacy/consent but are available from the corresponding author MK on reasonable request.

## Abbreviations

ART: Antiretroviral therapy
ABC: Abacavir
AZT: Zidovudine
3TC: Lamivudine
D4T: Stavudine
EFV: Efavirenz
FTC: Emtricitabine
LPV/r: Lopinavir/ritonavir
TDF: Tenofovir
BMI: Body mass index
COPD: Chronic obstructive pulmonary disease
DBP: Diastolic Blood Pressure
SBP: Systolic Blood Pressure
NCD: Non-communicable disease
LMIC: Low- and middle-income countries
