# Supplementary material for: Missed opportunities for NCD multimorbidity prevention in adolescents and youth living with HIV in urban South Africa
Source: BMC Public Health. 2020 Jun 1;20:821. doi: 10.1186/s12889-020-08921-0 (PMC7268240; doi:10.1186/s12889-020-08921-0)
Supplement: Supplementary file 1 — Additional file 1. contains detailed information on all data collected as part of the folder review process and definitions of variables used in the study. [file 12889_2020_8921_MOESM1_ESM.docx]

Additional File 1: Definition of key variables

| **HIV information** | |
| --- | --- |
| HIV Management: | CD4 levels and viral load information (most recent CD4 and viral load levels, date of last CD4 and viral load assessment).  Viral suppression was defined as undetectable viral load or viral load <20 copies/ml |
| Antiretroviral therapy (ART): | Age at initiation, ART start date, initial ART regimen, current ART regimens ranging from first line, second line and third line regimens |
| HIV opportunistic infections and any ART-related conditions: | Herpes zoster (shingles) |
|  | PPE- Pruritic Papular Eruption |
|  | Oral thrush and Oral candida |
|  | Oral hairy leucoplakia |
|  | Oesophageal candidiasis |
|  | PJP- Pneumocystis jiroveci pneumonia |
|  | HIV Encephalopathy |
|  | Cytomegalovirus (CMV) diseases (CMV- retinitis) |
|  | AZT-neutropenia |
|  | D4T-lipodystrophy, lipoatrophy |
| **NCD information** | |
| NCD diagnosis: | Diagnosis of diabetes, cancer, asthma, bronchitis, lung disease or any other COPD, depression, anxiety or other mental health condition |
| NCD treatment: | Chronic prescribed medications for diabetes, hypertension (e.g. Ciprofloxin, Hydrochlorothiazide, Enalapril maleate), epilepsy, asthma (e.g. Salbutamol metered dose inhaler, Ipratropium bromide), high cholesterol/ triglycerides (Statins), antipsychotics or anti-depressants (e.g. Risperidone, Valproate Sodium, Epilim), medication for bronchitis, lung disease or any other COPD |
| **NCD risk factors** |  |
| Overweight/obesity: | Body mass index (BMI) was calculated as the weight in kilograms divided by the square of the height in meters. Overweight defined as BMI ≥25 kg/m^2^ and BMI<30kg/m^2^; obese defined as BMI ≥30kg/m^2^. |
| Elevated blood pressure: | Systolic blood pressure (SBP) & diastolic blood pressure (DBP) according to South African hypertension practice guidelines:  Grade1/mild hypertension (SBP 140-159 or DBP 90- 99 mmHg), Grade 2/moderate hypertension (SBP 160-179 mmHg or DBP 100- 109 mmHg) or on treatment for elevated blood pressure |
| Smoking: | current smoker or history of smoking |
| Substance abuse: | current use or history of drug addiction, alcohol problem or other substance abuse |
| **General Medical Information** | |
| Family History: | Cancer, diabetes, tuberculosis, alcoholism, high blood pressure, epilepsy, or any other documented family history recorded in entire folder |
| Other conditions: | Pregnancy, epilepsy, learning difficulties, Failure to Thrive, trauma- injury and violence |
| Contraception: | Any prescribed contraception (Depo-provera or Nur-isterate) |
| **Other non-HIV infections** |  |
| non-HIV infectious disease diagnosis: | Tuberculosis, sexually transmitted infections (Herpes Simplex Virus), pneumonia, scabies |
| non-HIV infectious disease treatment: | Tuberculosis treatment (Rifampicin, Isoniazid, Pyrazinamide, Ethambutol), TB prophylaxis, STI treatment, Antibiotics, Antivirals, Steroids |
| **Health Promotion** | |
| **HIV-related** | Disclosure counselling prior to disclosure of HIV status;  Adherence counselling for issues with adherence or referral to community support group |
| **NCD-related** |  |
| Diet or healthy weight counselling: | Nutritional or healthy diet counselling or referral to a dietician |
| Substance abuse: | Alcohol, smoking or drug abuse counselling or referral to psychologist |
| Diabetes screening: | Fasting blood glucose or diabetes risk factor screen |
| Mental health counselling: | Referral to psychologist/ psychiatrist |
| Sexual and Reproductive Health: | Family planning, safe sex counselling, pap smear, breast examination; basic antenatal care counselling or infant feeding counselling for pre- or post-partum mothers;  Medical male circumcision |
| Other health promotion | Hygiene counselling; Physiotherapy/ Occupational Therapy |
